# The Oocyte/Antral Follicle Count Index: Does Maximizing Oocyte Yield Translate Into Better Assisted Reproductive Technology Outcomes?

**DOI:** 10.1002/jum.70211

**Published:** 2026-03-08

**Authors:** Shir Danieli‐Gruber, Onit Sapir, Dan Netanely, Avital Wertheimer, Avi Ben‐Haroush, Eran Altman, Tzippy Shochat, Yoel Shufaro

**Affiliations:** ^1^ Infertility and IVF Unit, the Helen Schneider Hospital for Women Rabin Medical Center – Beilinson Hospital Petah Tikva Israel; ^2^ Gray Faculty of Medical & Health Sciences Tel‐Aviv University Tel Aviv Israel

**Keywords:** IVF outcome, oocyte maturity, oocyte/AFC index

## Abstract

**Objectives:**

Whether aspiration of more oocytes than the antral follicle count (AFC) is beneficial for the current and future products of assisted reproductive technologies (ART) cycles.

**Methods:**

Data of the first oocyte aspiration for intracytoplasmic sperm injection performed between 2018 and 2022 in attempt to conceive (ICSI, *n* = 399) or for planned oocyte vitrification (fertility preservation [FP], *n* = 283) was retrieved. Each group was divided into 2 subgroups according to their “oocyte/AFC index” (OAFCI): <1 and ≥1.

Primary measures were clinical pregnancy rates (CPR) and live birth rates (LBR). Secondary measures were oocyte maturity rates, fertilization rates, and number of cryopreserved oocytes/embryos.

**Results:**

Oocyte maturity was unaffected by the OAFCI in the ICSI group, but was slightly lower in the FP group when OAFCI ≥1 (0.79 versus 0.84, *p* = .005). OAFCI ≥1 was associated with a higher number of cryopreserved embryos (ICSI, 3.9 versus 2.3, *p* < .001) and vitrified oocytes (FP, 13.4 versus 6.4, *p* < .001). The OAFCI had no impact on CPR (22.8% versus 28%, *p* = .27) nor LBR (19.8% versus 24.8%, *p* = .33). On multivariate analysis controlling for age, gonadotropin dosage, and duration of stimulation, OAFCI ≥1 was associated with a higher number of cryopreserved embryos (aOR 1.2, 95% CI 1.1–1.3, *p* < .001) in the ICSI group and higher vitrified oocytes (aOR 1.2, 95% CI 1.1–1.2, *p* < .001) and lower maturity index (aOR 0.2, 95% CI 0.05–0.75, *p* = .018) in the FP group.

**Conclusions:**

Aspirating more oocytes than the AFC does not influence the outcome of the fresh cycle but cryopreservation of more oocytes and embryos might translate into higher cumulative pregnancy rates later on.

**Abbreviations**
AFC: antral follicle count
AMH: anti‐Müllerian hormone
aOR: adjusted odds ratio
ART: assisted reproductive technologies
BMI: body mass index
CCCT: clomiphene citrate challenge test
CI: confidence interval
CPR: clinical pregnancy rate
FOI: follicle‐to‐oocyte index
FORT: follicular output rate
FP: fertility preservation
FSH: follicle‐stimulating hormone
GnRH: gonadotropin‐releasing hormone
hCG: human chorionic gonadotropin
ICSI: intracytoplasmic sperm injection
IRB: institutional review board
IU: international units
IVF: in vitro fertilization
LBR: live birth rate
LH: luteinizing hormone
MII: metaphase II
OAFCI: oocyte/antral follicle count index
OHSS: ovarian hyperstimulation syndrome
OPU: ovum pick‐up
OSI: ovarian sensitivity index
RCT: randomized controlled trial

Reproductive aging is a natural process of fertility decline as women progress from puberty to menopause.^1^ The rate of this decline varies widely among individuals of the same age^2^; for instance, two 30‐year‐old women could have significantly different reproductive lifespans. The decrease in fecundity is primarily due to the decline in both the quantity and quality of oocytes, along with hormonal changes such as increasing follicle‐stimulating hormone (FSH) and decreasing inhibin B and AMH levels.^3^ Ovarian reserve estimation uses indirect methods, including age, hormonal markers (FSH, inhibin B, AMH), dynamic tests (CCCT), and ultrasound antral follicle count (AFC).^4^ Among these, AFC stands out for its immediate results and predictive value for ovarian response, including predicting ovarian hyperstimulation syndrome (OHSS), making it superior to day‐3 FSH.^5^ AFC also correlates with embryo quality.^6^ A cut‐off of 3–4 follicles or retrieved oocytes is highly specific (73–100%) for predicting poor ovarian response and moderately specific (64–100%) for predicting failure to conceive, though its sensitivity is low (8–33%).^4^

Measuring ovarian reserve predicts ovarian stimulation response and determines the required FSH dose. Women with low ovarian reserve typically need higher FSH doses, while those with high reserve are at risk of OHSS.^7^ However, some women show ovarian responses inconsistent with traditional biomarkers like AFC and are categorized as poor or hyper‐responders. Alternative metrics such as follicular output rate (FORT), ovarian sensitivity index (OSI), and follicle‐to‐oocyte index (FOI) have been developed to better predict responses and optimize gonadotropin doses.^8^ FORT, introduced in 2011, is defined as pre‐ovulatory follicle count on hCG day divided by baseline AFC × 100.^9^ FOI represents the ratio of retrieved oocytes to AFC × 100, while OSI is calculated as recovered oocytes × 1000 divided by total FSH dose.^10^ These indexes were mainly investigated in the context of hyporesponders.^9^, ^11^

There is no data concerning the impact of the ratio between AFC and actual number of oocytes retrieved, “oocytes/AFC index” (OAFCI), on cycle outcome. The purpose of this study is to examine the association between OAFCI and the intermediate and final products of current and future assisted reproductive technologies (ART) cycles.

## Materials and Methods

### Setting and Patients

A retrospective cohort study in a tertiary university‐affiliated IVF medical center between 2018 and 2022. Data of 399 patients in their first oocyte aspiration for ICSI performed in attempt to conceive and 283 patients undergoing a first cycle of planned oocyte vitrification (fertility preservation [FP]) was retrieved. Patients not in their first cycle, natural cycle aspirations, and cases in which no oocytes were retrieved, were excluded. Each group (ICSI and FP) was divided into 2 subgroups according to their “oocyte/AFC index” (OAFCI): <1 and ≥1.

Stimulation protocol and FSH dose were determined according to the AFC and individual clinical consideration. The patients were triggered with Decapeptyl™ (Ferring, Germany) 0.2 mg and/or Ovitrelle™ (Merck, Switzerland) 50–250 mcg. The stimulation parameters, number of aspirated oocytes, their maturity rate, number of cryopreserved oocytes/embryos were compared between the OAFCI <1 and ≥1 subgroups for all patients. The clinical pregnancy (CPR) and live birth rates (LBR) were compared between the subgroups in the ICSI group.

### Data Collection

The study and control subgroups were compared for the following parameters: age, body mass index (BMI); gravidity, parity, infertility type (primary/secondary) and cause (mechanical, male, etc.), stimulation parameters (protocol, trigger type, total days of stimulation, total FSH [IU], LH [IU]), pre‐trigger blood levels of estradiol and progesterone, and endometrial thickness. The outcome measures were number of mature follicles, oocyte number, oocyte maturity index (for all groups), number of MII oocytes vitrified (FP group only), as well as fertilization index, number of cryopreserved embryos, CPR, early pregnancy loss, LBR (ICSI group only).

### “Oocytes/AFC Index” (OAFCI) Definition

The AFC was determined by professional sonographers using a 9 MHz transvaginal ultrasound probe in the early follicular phase most adjacent to the stimulation initiation in the absence of any ovarian cyst/mass/follicle 10 mm or larger. AFC was calculated by the sum of antral follicles with a mean diameter of 2–9 mm in the greatest 2‐dimensional plane, in both ovaries. The OAFCI was calculated as the ratio between the total number of oocytes retrieved at ovum pick‐up (OPU) and the AFC measured before the stimulation.

### Outcome Measures

The primary outcome measures were the CPR and LBR in the ICSI cycles in which embryo transfer was performed. Secondary outcome measures were differences in oocyte maturity rate, fertilization index, and number of cryopreserved oocytes (for the FP group) or the number of cryopreserved embryos (ICSI group).

CPR and LBR in the ICSI group were calculated only out of the cases in which embryo transfer was performed, or when there were no cryopreserved embryos. Cases in which embryo transfer was canceled, such as hyperstimulation, were excluded, and therefore narrowed the sample size from 399 to 297.

### Data Analysis and Statistics

Data were analyzed using SAS, version 9.4 (Cary, NC, USA). Continuous variables were analyzed by *t*‐test, and categorical variables by chi‐square or Fisher's exact test, as appropriate. Differences between groups were considered significant at a *p*‐value below .05. Parameters were compared between groups by univariate analysis followed by multivariate logistic regression analysis, adjusting for age, gonadotropin dosage, and duration of stimulation, considered potential confounders for the outcome of ART cycle.

### Ethical Approval

The study was approved by the local institutional review board (IRB) of Rabin Medical Center (RMC‐19‐0697). Informed consent was waived because of the retrospective design of the study and anonymous data collection and analysis.

## Results

A total of 682 women met the inclusion criteria and formed the final cohort, comprised of 399 first IVF cycle patients in an attempt to conceive in which ICSI was performed to all oocytes, and 283 patients undergoing planned oocyte vitrification (FP).

The baseline characteristics of the 2 groups are presented in Table 1. Mean age at aspiration was lower in women in the FP group when OAFCI ≥1 compared to OAFCI<1 (33.10 ± 5.56 versus 34.68 ± 4.82, *p* = .004), but not in the ICSI group (32.13 ± 5.16 versus 32.29 ± 5.51, *p* = .915). There were no between‐group differences in BMI, gravidity, parity, and infertility type in both ICSI and FP groups.

**Table 1:** Baseline Characteristics of the ICSI (*n* = 399) and FP (*n* = 283) Groups

Characteristics	ICSI			FP			
	OAFCI <1 (*n* = 200)	OAFCI ≥1 (*n* = 199)	*p* Value^a^	OAFCI <1 (*n* = 122)	OAFCI ≥1 (*n* = 161)	*p* Value^a^	
Age at aspiration (years)	32.29 ± 5.51	32.13 ± 5.16	.915	34.68 ± 4.82	33.10 ± 5.56	.004
Body mass index (kg/m^2^)	25.41 ± 5.84	25 ± 5.21	.521	22.93 ± 4.84	23.61 ± 4.68	.226
Nulliparity	169 (84.5)	178 (89.45)	.180	117 (95.90)	154 (95.65)	1
Infertility diagnosis						
Mechanical/Male	82 (41)	74 (37.19)	.473			
Other	118 (59)	125 (62.81)				
Infertility type						
Primary	105 (52.5)	108 (54.27)	.764			
Secondary	95 (47.5)	91 (45.73)				

ICSI, intracytoplasmic sperm injection; FP, fertility preservation; OACI, oocyte/AFC index. Values are presented as mean ± standard deviation for continuous variables and as number (percent) for categorical variables.

^a^ Normally distributed variables were compared using the *t*‐test, and non‐normally distributed variables using the Wilcoxon test.

The treatment characteristics of the 2 groups are presented in Table 2.

**Table 2:** Treatment Parameters of the ICSI (*n* = 399) and FP (*n* = 283) Groups

Characteristics	ICSI			FP		
	OAFCI <1 (*n* = 200)	OAFCI ≥1 (*n* = 199)	*p* Value^a^	OAFCI <1 (*n* = 122)	OAFCI ≥1 (*n* = 161)	*p* Value^a^
GnRH analog type						
Antagonist (Cetrotide/Orgalutran)	196 (98)	188 (94.47)	.071	120 (98.36)	157 (97.52)	.702
Agonist (Decapeptyl/Synarel)	4 (2)	11 (5.53)		2 (1.64)	4 (2.48)	
Trigger type						
Ovitrelle	19 (9.5)	24 (12.06)		8 (6.56)	6 (3.73)	
Decapeptyl only	21 (10.5)	41 (20.60)	0.010	38 (31.15)	58 (36.02)	.427
Dual trigger	160 (80)	134 (67.34)		76 (62.3)	97 (60.25)	
Total FSH dose (IU)	2343.19 ± 1066.50	2985.10 ± 1501.10	<.001	3869.80 ± 1308.92	3993.30 ± 1339.39	.208
Stimulation length (days)	9.08 ± 1.77	9.82 ± 2.05	<.001	9.08 ± 1.98	9.83 ± 1.78	<.001
Estradiol (pmol/L)	4304.89 ± 3042.11	6159 ± 4162.84	<.001	3488.64 ± 2985.05	4949.98 ± 3428.95.84	<.001
Progesterone (nmol/L)	1.6 ± 1.04	2.19 ± 1.15	<.001	1.96 ± 1.37	2.67 ± 1.50	<.001
LH (IU)	1.74 ± 1.65	1.65 ± 1.34	.916	1.62 ± 2.03	1.58 ± 2.00	.791
Endometrial thickness (mm)	9.8 ± 2.1	9.91 ± 2.68	.722	8.96 ± 2.28	9.16 ± 2.47	.390

ICSI, intracytoplasmic sperm injection; FP, fertility preservation; OACI, oocyte/AFC index. Values are presented as mean ± standard deviation for continuous variables and as number (percent) for categorical variables.

^a^ Normally distributed variables were compared using a *t*‐test, and non‐normally distributed variables using the Wilcoxon test.

In the ICSI group, total FSH dose used was significantly higher when OAFCI ≥1 compared to OAFCI<1 (2985.10 ± 1501.10 versus 2343.19 ± 1066.50, *p* < .001). Total stimulation days (9.82 ± 2.05 versus 9.08 ± 1.77, *p* < .001), estradiol (6159 ± 4162.84 versus 4304.89 ± 3042.11, *p* < .001) and progesterone levels (2.19 ± 1.15 versus 1.6 ± 1.04, *p* < .001) were significantly higher when OAFCI ≥1 compared to OAFCI<1. There were also higher rates of decapeptyl only and lower rates of dual trigger in the OAFCI ≥1 compared to OAFCI <1.

In the FP group, total stimulation days, estradiol, and progesterone levels were also significantly higher when OAFCI ≥1, compared to OAFCI<1 (9.83 ± 1.78 versus 9.08 ± 1.98, *p* < .001, 4949.98 ± 3428.95.84 versus 3488.64 ± 2985.05, *p* < .001, and 2.67 ± 1.50 versus 1.96 ± 1.37, *p* < .001, accordingly). No differences were found in GnRH analog and, LH and endometrial thickness between OAFCI ≥1 compared to OAFCI<1 in both ICSI and FP groups, and no differences in trigger type in FP only.

The treatment outcomes of the 2 groups are presented in Table 3. Oocyte maturity was unaffected by the OAFCI in the ICSI group, but was lower in FP group when OAFCI ≥1 (0.79 ± 0.19 versus 0.84 ± 0.18, *p* = .004). OAFCI≥1 was associated with a higher number of cryopreserved embryos (ICSI, 3.91 ± 4.53 versus 2.26 ± 2.99, *p* < .001) and a higher number of vitrified oocytes (FP, 13. 38 ± 9.80 versus 6.44 ± 5.03, *p* < .001). In the ICSI group, OAFCI had no impact on CPR (22.8% versus 28%, *p* = .270) and LBR (19.85% versus 24.84%, *p* = .331).

**Table 3:** Treatment Outcome of the ICSI (*n* = 399) and FP (*n* = 283) Groups

Characteristics	ICSI			FP		
	OAFCI <1 (*n* = 200)	OAFCI ≥1 (*n* = 199)	*p* Value^a^	OAFCI <1 (*n* = 122)	OAFCI ≥1 (*n* = 161)	*p* Value^a^
Oocytes number	10.65 ± 6.70	17.38 ± 9.78	<.001	7.86 ± 5.89	16.88 ± 11.19	<.001
Oocyte maturity rate (mature/total)	0.8 ± 0.2	0.8 ± 0.16	.169	0.84 ± 0.18	0.79 ± 0.19	.004
Mature vitrified oocytes		—		6.44 ± 5.03	13.38 ± 9.80	<.001
Fertilization index (fertilized/total)	0.76 ± 0.24	0.74 ± 0.20	.068		—	
Cryopreserved embryos	2.26 ± 2.99	3.91 ± 4.53	<.001		—	
Clinical pregnancy	45/161^b^ (27.9)	31/136^b^ (22.8)	.270		—	
Live birth	40/161^b^ (24.84)	27/136^b^ (19.85)	.331		—	

ICSI, intracytoplasmic sperm injection; FP, fertility preservation; OACI, oocyte/AFC index. Values are presented as mean ± standard deviation for continuous variables and as number (percent) for categorical variables.

^a^ Normally distributed variables were compared using *t*‐test, and non‐normally distributed variables, using Wilcoxon test.

^b^ The denominator contains only 297 cases in which embryo transfer was performed, or when there were no cryopreserved embryos. Cases in which embryo transfer was canceled, such as hyperstimulation, were excluded.

On post‐hoc multivariate analysis controlling for age, gonadotropin dosage and duration of stimulation, OAFCI ≥1 was associated with a higher number of cryopreserved embryos (aOR 1.2, 95% CI 1.1–1.3, *p* < .001) in the ICSI group and higher vitrified oocytes (aOR 1.2, 95% CI 1.1–1.2, *p* < .001) and lower oocyte maturity index (aOR 0.2, 95% CI 0.051–0.75, *p* = .018) in the FP group.

## Discussion

Ovarian responsiveness markers, such as FOI, OSI, and FORT have been utilized in matching the optimal dose of gonadotropins, exploiting the ovarian reserve and predicting better IVF outcomes thanks to their ability to grasp the developmental dynamics of follicles upon FSH stimulation.^11^

These calculated indexes were mainly investigated in the context of hyporesponders.^8^, ^11^ Less is known about the impact of such calculated indexes in normal responders, and the impact of eventually aspirating more or less oocytes than the “predicted” AFC (“oocytes/AFC index” OAFCI ≥1, or <1) on ART cycle outcome is still to be investigated.

Therefore, the aim of this study was to examine if aspiration of more oocytes than the AFC is beneficial for the intermediate and final products of current and future ART cycles. The rationale was to examine an index that reflects the “dynamic” nature of follicular growth in response to exogenous stimulation instead of the common ovarian reserve markers that represent a static snapshot only. We investigated this question in 2 scenarios: patients undergoing their first ICSI cycle, and patients undergoing their first oocyte vitrification cycle for FP. These scenarios were chosen because oocytes in both are denuded on OPU day, allowing maturity assessment near pick‐up time.

Data only from the first ever stimulation cycle was used in order to eliminate any effect of dosage adjustments in subsequent stimulations.

In the ICSI group, OAFCI ≥1 was associated with a higher number of available and cryopreserved embryos without a difference in the oocyte maturity rate. The OAFCI had no impact on the outcome of the fresh cycle in terms of CPR or LBR. In the FP group our principal finding was that a higher number of oocytes were vitrified when OAFCI ≥1, despite a lower maturity rate.

The parameters of the ovarian stimulation, its length, and peak Estradiol and Progesterone levels were higher when OAFCI ≥1 in both ICSI and FP groups. The total FSH dose used was higher only in the ICSI group. These results are not surprising and were reported before in other retrospective studies^12^ analyzing the association of follicle‐to‐oocyte index and clinical pregnancy in IVF treatment. A higher FOI was associated with a longer stimulation, aspiration of more oocytes and more mature oocytes, thicker endometrium, and more good quality embryos.

The OAFCI represents ovarian sensitivity to exogenous gonadotropins by taking into account both AFC and the number of oocytes retrieved. In a natural cycle, follicle selection occurs when the inter‐cycle FSH level surpasses the FSH threshold of the follicles with the highest FSH sensitivity,^13^ wherein IVF stimulation cycles, the circulating FSH levels are higher, resulting in stimulation of many other less sensitive follicles to develop. Higher circulating FSH levels in IVF stimulations can explain the higher number of MII oocytes, and also the small maturity rate due to the higher number of total oocytes retrieval.

FORT was also evaluated as a predictor for IVF‐ICSI outcome and demonstrated^14^ that a 1 unit rise in FORT increases significantly the number of oocytes retrieval per patient, number of metaphase II oocytes retrieved, and number of fertilized oocytes. Similarly, our study demonstrated higher oocytes number, higher number of cryopreserved embryos but the same oocytes maturity rate when OAFCI ≥1, in the ICSI group. It is not surprising that there were significantly more oocytes and cryopreserved embryos in the ICSI group when OAFCI ≥1, since mathematically OAFCI formula has “oocytes number” as its numerator. Nevertheless, the importance of these ovarian responsiveness markers stems from their ability to analyze the individual response to ovarian stimulation and therefore conclude when the FOI is low that follicles were not exploited fully, and offer other therapeutic options, such as FSH dose increment or LH addition, in order to optimize prognosis in the next ART cycles.^8^

A study investigating FORT, FOI and OSI as predictors for LBR in women of advanced reproductive age^11^ found an average LBR of 17.5%, but there was no association between the aforementioned indexes to LBR. Our study demonstrated a slightly higher LBR of 22%, but similarly, there was no association between OAFCI to LBR nor CPR in the fresh transfer cycle. Other studies reported that FORT values were higher in patients who succeeded to conceive in comparison to those who did not.^14^, ^15^ Since the number of oocytes retrieved and that of the available embryos is in positive correlation with the cumulative pregnancy and LBR,^16^, ^17^ we speculate that OAFCI ≥1 will eventually also translate into higher cumulative birth rates.

An explanation for similar or even lower LBR when a higher number of oocytes are retrieved could derive from higher estradiol levels that negatively affect the endometrium and implantation. Similarly, Sunkara (2011) showed a decline in LBR when aspirating more than 20 oocytes in the fresh cycle, leading toward a trend of milder ovarian stimulation.^18^

To check the stability of the association between OAFCI and the number of cryopreserved embryos in the ICSI group, the number of vitrified oocytes and oocyte maturity index in the FP group, we performed a multivariate analysis controlling for age, gonadotropin dosage, and duration of stimulation. These associations remained robust, suggesting there were no synergistic or antagonistic effects between OAFCI and these covariates.

The main strength of our study is the inclusion of a population from a single tertiary hospital with uniform treatment protocols. Our study differs from previous works by adjusting the results for a larger number of confounders rather than age only. The study is limited by its retrospective design and sample size. Another limitation is the lack of data on long‐term outcomes including cumulative pregnancy rates. Future prospective studies with larger sample sizes are needed to answer this question and thereby optimize ART treatment.

## Conclusion

Our results suggest that aspirating more oocytes than the AFC does not influence the outcome of the fresh cycle but harbors the possibility to cryopreserve more oocytes and embryos. This might translate into higher cumulative pregnancy rates later on. Additionally, this study is novel in investigating the relationship between the ultrasound‐based ovarian reserve testing, which is the most immediate and practical tool, and the early and delayed outcomes of ART.

## Data Availability

Data sharing not applicable to this article as no datasets were generated or analyzed during the current study.
